# The chromosome-level genome assembly of the dwarfing apple interstock *Malus hybrid* ‘SH6’

**DOI:** 10.1038/s41597-024-03405-x

**Published:** 2024-05-29

**Authors:** Jinrong Li, Huacheng Cai, Haixu Peng, Yulin Deng, Shijie Zhou, Ji Tian, Jie Zhang, Yujing Hu, Xiaoxiao Qin, Yuncong Yao, Yi Zheng, Sen Wang

**Affiliations:** 1 Beijing Key Laboratory for Agriculture Application and New Technique, College of Plant Science and Technology, Beijing University of Agriculture, Beijing, 102206 China; 2 Bioinformatics Center, Beijing University of Agriculture, Beijing, 102206 China; 3 Pomology Institute, Shanxi Agricultural University, Taigu, 030801 China; 4 Shanxi Key Laboratory of Germplasm Improvement and Utilization in Pomology, Taiyuan, 030031 China; 5 Ancient Tree Health and Culture Engineering Technology Research Center, National Forestry and Grassland Administration, Beijing, 100013 China

**Keywords:** Plant breeding, Genome

## Abstract

*Malus hybrid* ‘SH6’ *(M. honanensis × M. domestica*)is a commonly used apple interstock in China, known for its excellent dwarfing characteristics and cold tolerance. In this study, a combined strategy utilizing PacBio HiFi, Hi-C and parental resequencing data were employed to assemble two haploid genomes for ‘SH6’. After chromosome anchoring, the final hapH genome size was 596.63 Mb, with a contig N50 of 34.38 Mb. The hapR genome was 649.37 Mb, with a contig N50 of 36.84 Mb. Further analysis predicted that repeated sequences made up 59.69% and 62.52% of the entire genome, respectively. Gene annotations revealed 45,435 genes for hapH and 48,261 genes for hapR. Combined with genomic synteny we suggest that the hapR genome originates from its maternal parent *M. domestica* cv. Ralls Janet, while the hapH genome comes from its paternal parent, *M. honanensis*. The assembled genome significantly contributes to the discovery of genes associated with apple dwarfing and the molecular mechanisms governing them.

## Background & Summary

Apple (*Malus spp*.) is the most abundantly cultivated fruit tree of temperate regions^1^. Apple cultivation covers extensive acreage and has high economic value, ranking as the second most produced fruit in China, only surpassed by citrus. Similar to other fruit trees, apple has high genetic heterozygosity and is mainly reproduced by grafting to maintain fine cultivar properties. In apple production, dwarf rootstock use for high-density planting not only enhances apple yields but improves fruit quality. In 1978, the Shanxi Fruit Research Institute hybridized and bred the SH series through crossbreeding *M. domestica* cv. Ralls Janet and *M. honanensis*. These rootstocks incorporated the advantages of both parental lines. Due to its excellent cold resistance, compact height (c.1.8 m), graft compatibility, and high yield, ‘SH6’ is widely used in mountainous areas such as Beijing^2–4^.

With rapid sequencing technology advances, multiple haploid genomes have been successfully assembled in *Malus*, but the materials used typically originate from varieties outside of rootstocks. Due to relatively high heterozygosity and significant differences among *Malus* species genomes, it is crucial to construct haploid genomes for the studied species as they form the basis for analyzing homologous chromosome expression dominance. To facilitate effective breeding of new apple rootstocks and dwarf varieties, assembling a high-quality genome for this rootstock is a crucial step.

**Fig. 1:**
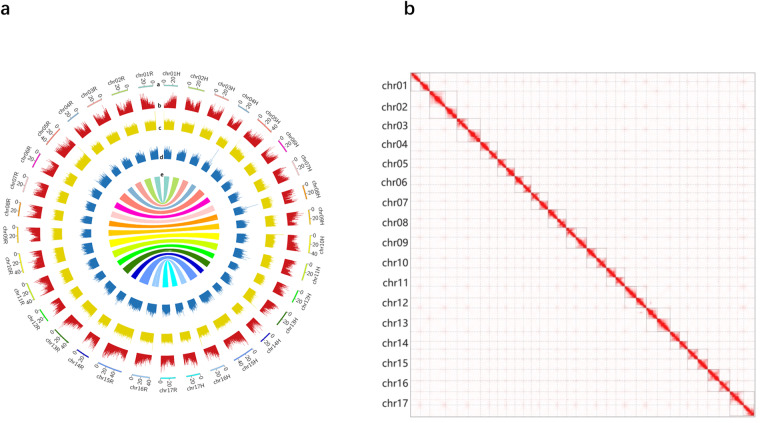
Genome-wide Circos map and Hi-C heat map in *M. domestica* ‘SH6’. (**a**)The circus map of *M. domestica* ‘SH6’. (a-Chromosome ID and length, b-Density of protein-coding genes, c-Density of LTR elements, d-GC content, e-Paralog synteny relationships) (**b**) The plot displays sequences exclusively anchored on chromosomes. Solid line boxes represent the 17 chromosome pairs, while dashed line boxes indicate haplotype-resolved genomes.

Using combined Pacbio HiFi and parental resequencing data, we assembled a high-quality genome for ‘SH6’, based on *K-*mer analysis, with a repeat rate of 52.6% and heterozygosity of 3.58%. Assembly results yielded a hapH genome of 606.93 Mb with a contig N50 of 34.38 Mb, and after chromosomal anchoring, the final genome was 596.63 Mb. The hapR genome was 662.26 Mb with a contig N50 of 36.84 Mb, and the final genome was 649.37 Mb.Telomere analysis revealed that hapH and hapR had 10 and 12 chromosomes with double-ended telomeres, and 7 and 5 chromosomes with single-ended telomeres. Gene annotations identified 45,435 genes for hapH and 48,261 genes for hapR (Fig. 1a). Using BUSCO analysis, hapH achieved a completeness score of 98.6%, while hapR reached 99.0%. This study provides valuable insights for dwarfing rootstock breeding and molecular mechanisms.

## Methods

### Plant material

On April 11, 2021, at the Shanxi Agricultural University (formerly Shanxi Academy of Agricultural Sciences) Fruit Research Institute in Taigu, Shanxi, we collected one-year-old shoot tips and leaves from the original ‘SH6’, Ralls Janet and *M. honanensis* trees. On April 24, 2023, we collected flowers, leaves and young stems of the same ‘SH6’.

### DNA extraction and Genome sequencing

Genomic DNA extraction from ‘SH6’ leaf tissues was conducted using a Plant Genomic DNA Extraction Kit (CTAB) following the manufacturers protocol. The construction of PacBio SMRTbell libraries was accomplished using a SMRTbell® Express Template Prep Kit 2.0 (Pacific Biosciences, PN 101-853-100) and involved the following steps: (1) Evaluation of 10 μg of gDNA, with library construction performed when DNA fragments exceeded 40 kb; (2) Qualifying DNA fragments were sheared into 15 kb fragments using a Megaruptor instrument (Diagenode B06010001) and DNA concentration was determined using AMPure®PB Beads (Pacific Biosciences 100-265-900); (3) A SMRTbell library was constructed according to the kit’s instructions, involving the removal of single-stranded overhangs, DNA damage repair, end repair, A-tailing, adapter ligation, and enzymatic digestion. Fragment selection was performed using a SageELF (Sage Science ELF000). We sequenced the PacBio library on the PacBio Sequel II system, generating ~19.66 Gb of clean data (~30×).

DNA extracted from leaves of Ralls Janet and *M. honanensis* were measured using Qubit fluorescence photometer for DNA concentration and 1% agarose gel electrophoresis for DNA integrity. Then, the Watchmaker DNA Library Prep Kit with Fragmentation (CAS:7K0019-096) was used to prepare the library. 200 ng was taken from each DNA sample for library preparation, in which the DNA was enzymtically digested into fragments averaging 350 bp. After the library was constructed, Qubit3.0 was used for preliminary quantification, and then fragment analyzer was used to detect the fragment size distribution. After the size and peak distribution of the library met the expectations, qPCR quantification was performed using ABI Quant Studio 12 K Flex to ensure the quality of the library. Finally, The cluster generation and sequencing were performed on Illumina Novaseq 6000 S4 platform, using NovaSeq 6000 S4 Reagent kit V1.5. and the double-ended sequencing program (PE) was run to obtain 150 bp double-ended sequencing reads. For Ralls Janet and *M. honanensis*, ~25.51 Gb and ~24.00 Gb data are generated, respectively.

For the Hi-C library, we used formaldehyde for crosslinking cells, thereby maintaining both intra- and intermolecular interactions, and preserving the cell’s 3D structure. Following crosslinking, we used the restriction enzyme HindIII for DNA digestion and incorporated biotin-labeled nucleotides during the end repair stage. Then proteins were digested at the ligation junctions to release DNA from the cross-linked state. DNA was purified and fragmented enzymatically, marked for DNA capture, and constructed into sequencing libraries. The Hi-C library, sequenced on the Illumina NovaSeq 6000 (PE150), produced ~90.44 Gb of clean data.

### RNA extraction, library preparation and sequencing

Mixed sample including flowers, leaves, stems were collected from the ‘SH6’. Total RNA was isolated by standard TRIzol protocol. The integrity of the RNA was determined with the Agilent 2100 Bioanalyzer (Agilent Technologies, Palo Alto, California, USA). After the sample was qualified, 3 ug total RNA was used as the starting material to construct a transcriptome sequencing library. RNA concentration of library was measured using Qubit® RNA Assay Kit in Qubit® 3.0 to preliminary quantify and then dilute to 1 ng/μl. Sequencing was performed using the Illumina platform, and ~20.78 Gb paired-end reads were obtained.

### Genomic survey and analysis

‘SH6’ genome size, heterozygosity, and ploidy assessment were evaluated using Jellyfish^5^ and Genomescope2^6^ based on the *K*-mer approach. At *K*-mer = 21, the estimated genome size of ‘SH6’ repeat rate was 52.6% and heterozygosity of 3.58%. Furthermore, the ploidy analysis results suggested that the probability of ‘SH6’ being a diploid apple interstock was 0.71, indicating that it has a highly heterozygous diploid genome.

### Genome assembly

**Fig. 2:**
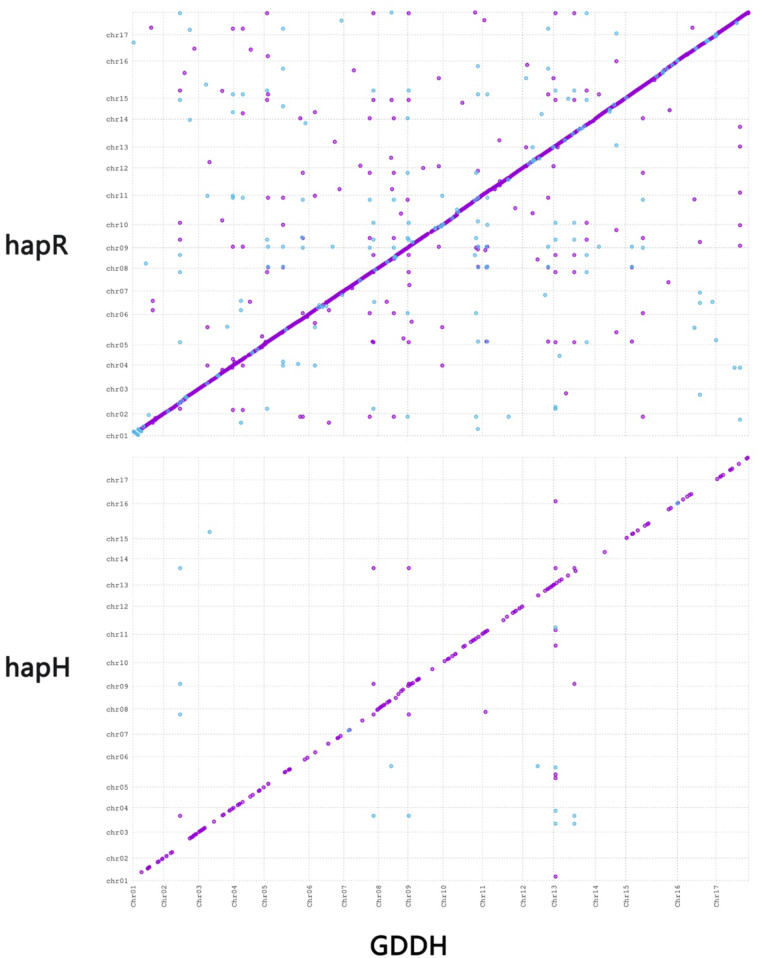
Sequence collinearity between *M. domestica ‘SH6’* hap1 and hap2 with ‘Golden Delicious’ genome. Purple indicates the sequence is in the same direction as the reference sequence, while blue represents the opposite direction.

Initial assembly of the ‘SH6’ apple interstock genome was conducted in the Hifiasm^7^ (v0.19.6) using tiro-binning mode with parental resequencing data (−1 pat.yak −2 mat.yak), resulting in two single-haplotype contigs. Based on the Hi-C interaction data obtained from 3D-DNA^8^, we conducted clustering, ordering, and correction to accurately anchor the contigs onto the 34 chromosomes. Subsequently, manual refinement was performed using Juicerbox^9^ to ensure precision and integrity of the final chromosome assembly, addressing locations, inversions, and chromosome boundaries. The final haplotype-resolved genomes (hap1 and hap2) were obtained by the ‘agp2fa’ mode of RagTag^10^. Then, MUMMER^11^ (v4.0) was used to compare the two genomes with the ‘Golden Delicious’ genome. The result showed that all chromosomes of hap1 were higher similary with the ‘Golden Delicious’ genome than hap2 (Fig. 2), indicting hap1 and hap2 origined from *M. honanensis* and ‘Ralls Janet’, respectively, defining as hapH and hapR.

**Fig. 3:**
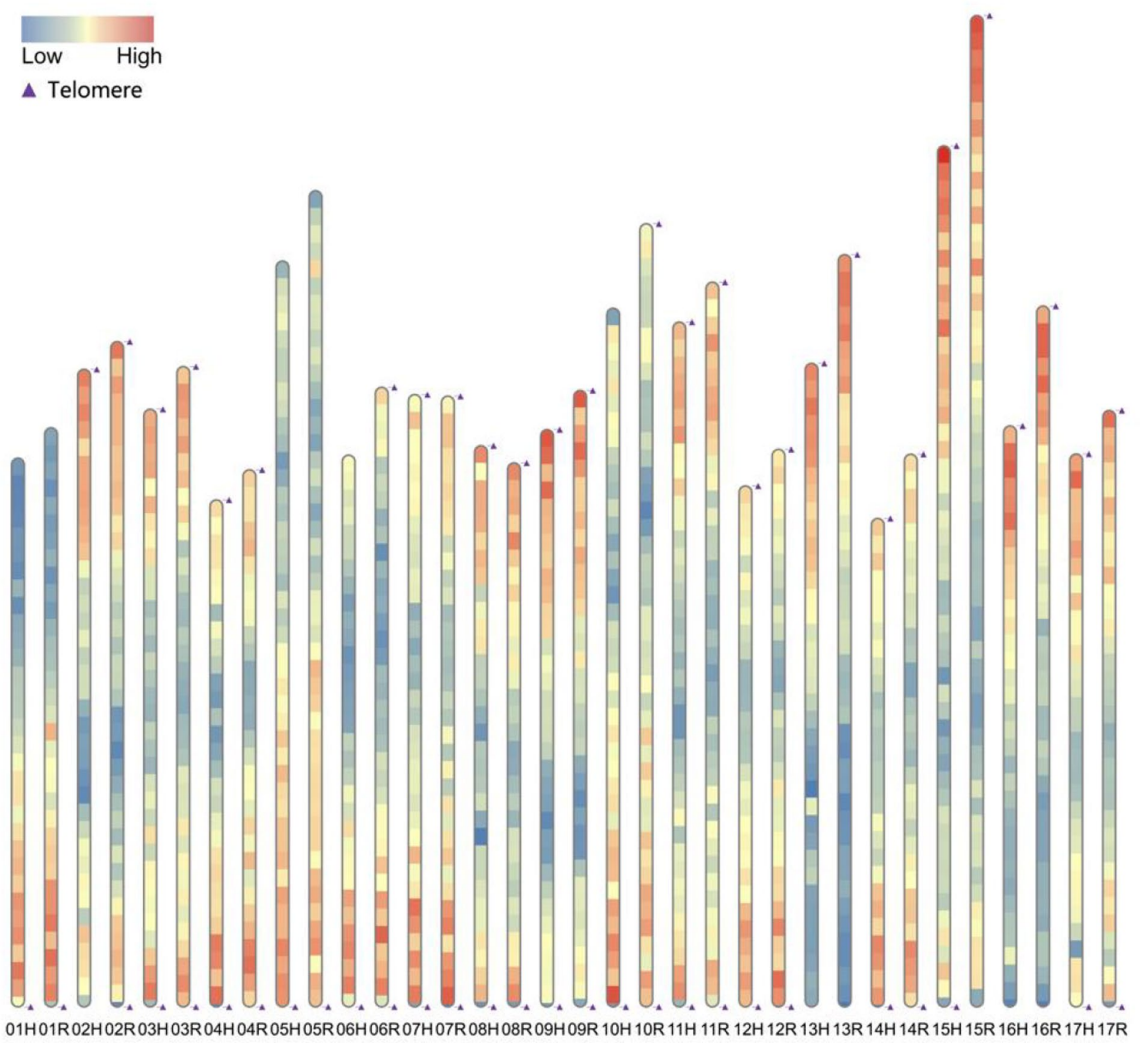
Chromosome telomere and gene density map of *M. domestica* ‘SH6’. Red represents high-density regions of genes, while blue signifies low-density areas. The higher the chromosome, the larger the size it indicates.

‘SH6’ hapH genome initial assembly size was 606.93 Mb with a contig N50 of 34.38 Mb, and final genome size after chromosomal anchoring was 596.63b. ‘SH6’ hapR genome initial assembly size was 662.26 Mb with a contig N50 of 36.84 Mb, and final genome size after chromosomal anchoring was 649.37 Mb. Compared with ‘Golden Delicious’ and ‘Gala’ genomes, ‘SH6’ was better in assembly integrity and busco assessment (Table 1). To further confirm integrity of the two haploid genomes, TRF was used for Telomere analysis, which revealed that hapH and hapR had 10 and 12 chromosomes with double-ended telomeres, and 7 and 5 chromosomes with single-ended telomeres, respectively. Telomere detection indicated high genome integrity in the assembled genome (Fig. 3).

**Table 1:** Genome assembly of ‘SH6’.

	hapH	hapR	Golden Delicious	Gala_haploid
Assembled contigs size (Mb)	606.93	662.26	703.0	—
Chromosome genome(Mb)	596.63	649.37	625.3	657.7
Contig N50(Mb)	34.38	36.84	37.6	38.0
Unanchored size(Mb)	10.3	12.89	77.7	—
Anchoring rate(%)	98.3	98.05	88.9	—
Complete Busco(%) (C)	98.6	99.0	98.0	97.4
Complete and single-copy BUSCOs (S)	59.4	62.3	62.3	62.3
Complete and duplicated BUSCOs (D)	39.2	36.7	35.7	35.1
Missing (M)	0.6	0.4	1.3	1.9
Frangments (F)	0.8	0.6	0.7	0.7
Gaps	56	20	4092	14330

### Genome annotation

Genome annotation mainly includes repeated sequence annotation, gene structure and gene function annotations, of which repeated sequence annotation is the first step. To analyze ‘SH6’ repeated sequences, we employed both *de novo* prediction and homology-based methods. RepeatModeler^12^ (v1.0.11) was used for *de novo* prediction of repetitive sequences, while RepeatMasker^13^ (v4.1.2) was used to identify transposable elements through homology-based methods. Additionally, TRF^14^ (v4.09) was utilized to annotate tandem repeat sequences in the genome. Transposons in ‘SH6’ hapH genome accounted for approximately 59.69% of the entire genome, while transposons in the hapR genome made up approximately 62.52% of the total genome, with the majority located at the terminal ends (Table 2).

**Table 2:** Summary statistics of transposable elements(TE) annotated in the ‘SH6’ genome of Apple Rootstock.

Type	hapH			hapR		
	Number	Length(bp)	Percentage of Genome(%)	Number	Length(bp)	Percentage of Genome(%)
Retrotransposon	302007	219,865,359	36.85	332293	270,237,467	41.62
LINEs	12,517	6,946,968	1.16	17,046	19,526,510	3.01
LTR elements	289490	212,918,391	35.69	315247	250,710,957	38.61
Ty1/Copia	105708	81,340,243	13.63	118411	93,318,235	14.37
DNA	14,697	9,427,863	1.58	16,938	9,870,510	1.52
Unclassified	491613	126,815,565	21.26	482420	125,874,628	19.38
Total interspersed		356108787	59.69		405,982,605	62.52

After masking repetitive sequences, protein-coding genes in the apple interstock ‘SH6’ were identified through an integrated approach involving *de novo* prediction, transcriptome data, and homologous protein alignments^15^. The workflow comprised three key steps. Firstly, a homology-based prediction strategy was employed, where 5 previously sequenced apple genome protein sequences (*M. sylvestris*, ‘Golden Delicious’, ‘Gala’, *M. baccata* and *M. sieversii*) were selected. Exon regions and splice sites were determined by sequence alignment based on protein homology. In the second step, *ab initio* prediction was performed, where Augustus^16^ (v3.4) was used to generate training models based on the complete sequences of 68,622 genes identified in the previous homology-based prediction. Step three involved auxiliary annotation using ‘SH6’ transcriptome data from mixed flowers, leaves and stems. HISAT2^17^ (v2.2) and StringTie^18^ (v1.3) were used for transcript assembly to predict splice sites and candidate exon regions. Finally, the results from the three strategies were integrated using Maker^19^ (v3.1) to produce the final annotation file. Final results of protein-coding gene prediction revealed 45,435 and 48,261 genes in the hapH and hapR genomes, respectively (Table 3).

**Table 3:** Statistics of gene models of ‘ SH6’ haplotype genomes.

	hapH	hapR	Golden Delicious	Gala_haploid
Gene number	45,435	48,261	45,116	46,165
CDS	202,881	214,311	215,867	250,069
mRNA	45,435	48,261	45,116	46,165
Intron GC content (%)	37.20	37.40	38.00	38.00
Complete Busco(%) (C)	95.0	95.9	96.7	94.4
Complete and single-copy BUSCOs (S)	62.5	64.5	65.1	61.3
Complete and duplicated BUSCOs (D)	32.5	31.4	31.6	33.1
Missing (M)	4.2	3.0	1.4	3.9
Frangments (F)	0.8	1.1	1.90	1.70

Subsequently, functional annotation of the protein-coding genes was performed. Protein sequences were aligned against known public databases (Arabidopsis protein database, GenBank Nr, SwissProt^20^, TrEMBL, and) using Diamond^21^ (v2.0). InterProScan^22^ (v5.59) was used to identify functional protein structural domains, as well as Gene Ontology (GO) terms. Protein sequences were compared with the Kyoto Encyclopedia of Genes and Genomes (KEGG) database using KAAS (https://www.genome.jp/tools/kaas/) to identify gene functions and associated metabolic pathways. AHRD^23^ (v3.3) was used to integrate results from SwissProt, TrEMBL, and Arabidopsis protein database alignments to predict more comprehensive and accurate gene functions. Through comparisons with SwissProt, AHRD, Nr, and Arabidopsis databases, hapH and hapR annotated to 30,190 and 32,005, 39,180 and 41,143, 43,160 and 45,157, 34,315 and 36,338 genes, respectively (Table 4).

**Table 4:** ‘SH6’ functional annotation of genes.

	hapH		hapR	
	Gene number	Percent (%)	Gene number	Percent (%)
GenBank-NR	43,160	94.99	45,157	93.57
A. thaliana	34,315	75.53	36,338	75.29
SwissProt	30,190	66.45	32,005	66.32
TrEMBL	39,180	86.23	41,143	85.25
KEGG	20,698	45.56	22,201	46.00
GO	19,411	42.72	20,488	42.45
TF/TR	3,002	6.61	3,138	6.50
PK	1,562	3.44	1,664	3.45

## Data Records

The genomic sequencing HiFi data from PacBio Sequel II system, generating ~19.66 Gb data(~30x) have been deposited in the NCBI Sequence Read Archive (SRA), with accession numbers SRR26558196^24^ and SRR26558195^24^. The Hi-C sequencing data from Illumina NovaSeq 6000 platform, produced ~90.44 Gb (~150x) of clean data is also stored in the NCBI SRA, with accession numbers SRR26558194^24^. The transcriptome sequencing data of Ralls Janet and *M. honanensis*, from NovaSeq 6000 platform, generating ~25.51 Gb (~42x) and ~24.00 Gb (~40x) data are also stored in the NCBI SRA, with accession numbers SRR27432230^24^ and SRR27432231^24^, respectively. The chromosomal assembly and dataset of gene annotation have been deposited at Figshare(10.6084/m9.figshare.24941565)^25^. The assembly genomes files of ‘SH6’ were stored under the accession GCA_036324465.1^26^ and GCA_036324445.1^27^.

## Technical Validation

Genome assembly quality assessment: As portrayed in the heatmap, genome assembly accuracy was characterized by relatively independent Hi-C signals observed between the 17 pairs-chromosomes (Fig. 1b). Utilizing parental resequencing data, we partitioned the haploid genomes. The assessment involved computing the unique read ratio for individual chromosomes mapped on hapH and hapR to validate their consistency. BWA-MEM^28^ algorithm was used to map the parental resequencing data against the two haplotype-genomes. Unique reads were subsequently extracted to calculate the ratio (RH:RR), where RH and RR denote the percentage of unique reads mapped to the hapH and hapR genomes, respectively. The ratio derived from *M. honanensis* resequencing data was approximately 91:9, whereas for *M. domestica* cv. Ralls Janet resequencing data, the ratio was 13:87. These findings serve to validate the accuracy of the assembly (Table 5).

**Table 5:** The ratio of uniq reads from parental resequencing data mapped on HapH and HapR.

Chromosome	M. honanensis			M. domestica cv. Ralls Janet		
	# HapH	#HapR	Ratio	# HapH	#HapR	Ratio
chr01	1,464,457	95,794	94:6	121,994	1,192,940	9:91
chr02	1,752,505	172,780	91:9	341,759	1,303,167	21:79
chr03	1,542,130	141,429	92:8	177,227	1,325,517	12:88
chr04	1,404,918	100,179	93:7	158,206	1,144,954	12:88
chr05	1,955,226	233,874	89:11	257,833	1,688,721	13:87
chr06	1,427,027	107,505	93:7	129,106	1,416,708	8:92
chr07	1,703,765	112,771	94:6	263,238	1,261,259	17:83
chr08	1,579,938	101,229	94:6	267,456	1,122,376	19:81
chr09	1,600,686	112,858	93:7	217,874	1,143,901	16:84
chr10	1,813,710	164,959	92:8	205,144	1,710,004	11:89
chr11	1,787,542	346,736	84:16	277,715	1,528,933	15:85
chr12	1,398,305	105,904	93:7	167,240	1,197,537	12:88
chr13	1,787,135	94,329	95:5	215,640	1,344,834	14:86
chr14	1,285,163	78,900	94:6	125,156	1,199,491	9:91
chr15	2,578,728	174,994	94:6	416,878	1,926,310	18:82
chr16	1,645,390	307,996	84:16	144,394	1,621,325	8:92
chr17	1,466,407	135,775	92:8	194,641	1,325,420	13:87

**Fig. 4:**
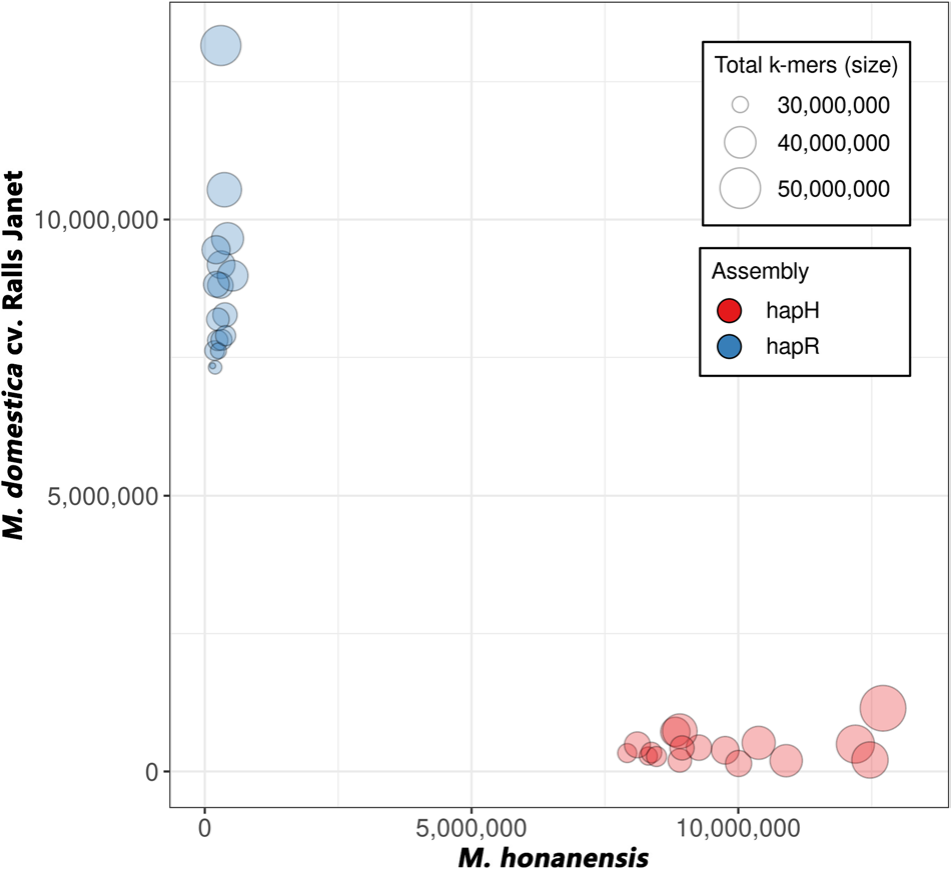
The hap-mer blob plot to demonstrate the haplotype effect of ‘SH6’genome. Each circle represents a chromosome, hapH is closer to *M. domestica* cv. Ralls Janet, and hapR is all closer to *M. honanensis*, symmetrical on both sides of the diagonal.

Evaluation phasing process was performed using Merqury^29^ software based on the k-mer spectrum of the parental and the SH6 genomic reads. As depicted in Fig. 4, fewer hap-mers in hapH were found from the *M. domestica* cv. Ralls Janet, while the other hapR was entirely *M. domestica* cv. Ralls Janet, the closer proximity of the circle to the axis indicates that better the phasing performance is.

Genome quality was assessed using BUSCO^30^, resulting in a remarkable completeness score of 98.6% for hapH and an even higher 99.0% for hapR. Notably, 59.4% and 62.3% of single-copy BUSCOs were identified in hapH and hapR, respectively, underscoring the high-quality nature of the assemblies.

## Data Availability

There is no custom code was used during this study.The public softwares used in this work, were cited in the Methods section. If no detail parameters were mentioned for a software, default parameters were applied with the guidance.
